# The placenta and adverse pregnancy outcomes – opening the black box?

**DOI:** 10.1186/1471-2393-15-S1-A5

**Published:** 2015-04-15

**Authors:** Alexander Heazell

**Affiliations:** 1 Maternal and Fetal Health Research Centre, Manchester Academic Health Science Centre, University of Manchester, UK

## 

A healthy placenta is critical for a healthy pregnancy. Conversely, abnormal placental structure and function is seen in conditions which are associated with stillbirth including: fetal growth restriction, preeclampsia, placental abruption and obstetric cholestasis. Abnormalities can be seen ranging from a reduction in placental size in stillbirth to microscopic changes in placental villous architecture [1]. Placental examination is advocated after stillbirth by respected guidelines [2-4]; this recommendation is based upon the frequency of abnormalities seen in placentas after stillbirth [5,6], the reduction in unexplained stillbirths when placental histological examination is performed and the cost-effectiveness per abnormality detected [1,7].

The placenta has previously been referred to as a “diary of pregnancy” and it is tempting to compare examination of the placenta after stillbirth with the “black-box” flight data recorder used after aircraft accidents. To be certain that placental findings are significant in a case of stillbirth they should reflect (relevant) changes that occurred prior to fetal death. Thus, there should be no artefact from in-utero retention or storage. Placental findings should give information regarding conditions present and be specific for adverse pregnancy outcome (i.e. not occur in healthy pregnancy). Ultimately, the information obtained must be useful, aiding understanding of death by clinicians and inform future care.

Storage and fixation of placental tissue can alter findings on examination. Naeye et al. states that “troublesome artefacts” can appear after 48 hours of refrigeration [8]. This is supported by qualitative and quantitative assessment Garrod et al. demonstrated changes in villous vascularity after 48 hours refrigeration [9]. Thus, every effort should be made to minimise the time of storage prior to examination. The effects of retention *in utero* before birth are more difficult to assess as the time of fetal death is usually unknown. Genest estimated that *in utero* retention was associated with villous degeneration, particularly of fetal blood vessels and villous stroma [10].

A systematic review of histopathological assessment of the placenta found that a placental cause is reported in 11.2 - 64.9% and associated with stillbirth in 31.5% - 84% of cases [11]. The greatest influence on the proportion of stillbirths classified as having “placental” abnormalities was the classification system employed. The specificity of placental abnormalities for stillbirth has previously been questioned by the high incidence of histological lesions in apparently normal pregnancies and the large variation in agreement between pathologists when identifying lesions (Kappa – 0.25-0.91) [12,13]. These data highlight the importance of international consensus in the definition of placental lesions to improve study quality. Accurate description of lesions will also enable better understanding of their origins. One example of this is syncytial knots (also known as syncytial nuclear aggregates). The formation of syncytial knots are increased in hypoxia and oxidative stress *in vitro*[14], which supports the reported association between syncytial knots/nuclear aggregates and maternal vascular malperfusion [13,15].

Furthermore, evaluation of placental structure and function can be used to explore clinical scenarios relating to stillbirth such as maternal perception of reduced fetal movements, advanced maternal age and fetal growth restriction [16-18]. These clinical conditions are all associated with alterations in placental structure, specifically increased syncytial knots/nuclear aggregates, changes in trophoblast proliferation and alterations in amino-acid transport [14,19-21]. Importantly, these observations provide plausible biological association between these clinical scenarios and stillbirth from placental causes. These suggest that better appreciation of placental function *in utero* may provide an opportunity to identify pregnancies at risk of stillbirth to target intervention [22,23].
